# Overview of Methods for Assessing Antimicrobial Use in Outpatient Settings in High-Income Countries: A Narrative Review

**DOI:** 10.3390/antibiotics14111161

**Published:** 2025-11-16

**Authors:** Anita Kotwani, Mihir Chauhan, Elizabeth Roughead, Arno Muller, Martina Escher, Benedikt Huttner, Verica Ivanovska

**Affiliations:** 1 Vallabhbhai Patel Chest Institute, University of Delhi, Delhi 110007, India; anitakotwani@gmail.com; 2 Department of Pharmacology, Lady Hardinge Medical College, New Delhi 110001, India; mihir.docmusic@gmail.com; 3 Clinical and Health Sciences, University of South Australia, Adelaide 5000, Australia; libby.roughead@unisa.edu.au; 4 Antimicrobial Resistance Department, World Health Organization, 20, Avenue Appia, 1211 Geneva, Switzerland; amuller@who.int (A.M.); escherm@who.int (M.E.); bhuttner@who.int (B.H.)

**Keywords:** antimicrobial, use, outpatients, AWaRe, high-income countries (HICs)

## Abstract

**Background/Objectives**: Over 80–90% of antibiotics are used in outpatient settings, where interactions among diverse prescribers, dispensers, and patients create complex usage patterns. The study examines how outpatient antimicrobial use is monitored in high-income countries (HICs), focusing on data sources and their relevance for stewardship and surveillance. **Methods**: This narrative review searched MEDLINE and Embase for English-language studies reporting outpatient antimicrobial use in HICs (from inception to 2023), supplemented by reference screening, targeted Google web searches, and expert input. Studies were categorized by data collection method, study period, and WHO Region. Key characteristics such as patient group, disease focus, country, dataset, and reported outcome measures were also extracted and tabulated. **Results**: We identified 287 studies, of which 79 met inclusion criteria. Most (*n* = 76) were conducted after 2000 and spanned all four WHO regions with HICs. Of the 73 studies using surveillance databases, six types were identified: dispensing (*n* = 13), health insurance (*n* = 24), GP prescribing (*n* = 17), commercial (*n* = 9), procurement (*n* = 3), and multinational networks (*n* = 7). Six studies used surveys: general point prevalence (*n* = 1), indication-specific audits (*n* = 3), patient exit interviews (*n* = 1), and community surveys (*n* = 1). Common outcome measures included DID, Days of Therapy, and patterns of antimicrobial use by season, age, sex, indication, and prescriber. Of the 48 studies published after 2018, nine reported using AWaRe metrics. **Conclusions**: Surveillance databases were the main method for tracking outpatient antimicrobial use in HICs; surveys were less common. Antibiotic use has increasingly been reported by AWaRe category, especially in prescribing and insurance studies. Further indicators and tech-driven tools are needed to support stewardship.

## 1. Introduction

Antimicrobial resistance (AMR) is a global public health threat that not only undermines the management of infectious disease but compromises the efficacy of modern medicine [1]. The inappropriate use of antimicrobials contributes to emergence and spread of AMR [2]. In 2015, the World Health Organization (WHO) established the Global Antimicrobial Resistance and Use Surveillance System (GLASS) as part of the Global Action Plan on AMR to support standardized surveillance of AMR and antimicrobial use (AMU) [3]. As of 2025, 74 countries have provided national data on antimicrobial use to WHO GLASS module for antimicrobial use (GLASS-AMU) for the period 2016–2022 [4]. In 2017, WHO introduced the AWaRe as a cornerstone of antimicrobial stewardship efforts, classifying antibiotics into Access, Watch, and Reserve (AWaRe) categories based on their spectrum of activity and potential to develop resistance [5,6]. The AWaRe framework has since been used to optimize antibiotic use and support the development of quality improvement measures against AMR.

Over 80% to 90% of antimicrobial use takes place in outpatient settings, highlighting the need to not neglect antimicrobial stewardship in this sector [7]. Prescription of antibiotics for viral infections or use of broad-spectrum Watch antibiotics when Access antibiotics would suffice is common [8]. Examining and adhering to regulations, policies, clinical guidelines, and strategies can improve the appropriateness of antimicrobial use [9]. Data on antimicrobial use in outpatient settings can inform the development and assessment of interventions.

In many high-income countries (HICs), antimicrobial use is routinely monitored through national or subnational data health information systems that collect longitudinal data on medicines utilization. The data sources comprise healthcare databases that capture standardized data year-round at various stages of the life cycle of medicines, including wholesale distribution, prescribing, dispensing, or reimbursement. Initially set up for administrative, financial, pharmacoepidemiologic or other research purposes, these routine surveillance systems can provide medicine-level data for secondary analyses on the types and quantities of antimicrobials used in hospital and outpatient settings [10,11,12,13,14,15]. In addition to surveillance, surveys focusing on prescribers, dispensers, and community members can collect antimicrobial use data in outpatient settings, generating clinical-level data with insights into prescribing indications, patient demographics, and adherence patterns.

These diverse data sources offer valuable insights into outpatient antimicrobial use in HICs. Strengthening surveillance systems is essential to improving antimicrobial use and achieving customized targets. WHO initially set a benchmark of 60% for national antibiotic use from the Access group to promote stewardship and comparability. This target was subsequently strengthened to 70% in the 2024 UNGA commitment, signaling the need for accelerated progress and reflecting that most antibiotic use occurs in primary care, where common infections can be treated with Access antibiotics. [16] To support this goal, we conducted a narrative review to learn about existing data collection methods for antimicrobial use in outpatients in high-income countries. This review, along with our separate ongoing review focused on low- and middle-income countries (LMICs), will inform a forthcoming WHO guidance document on monitoring antimicrobial use in PHC.

## 2. Methods

### 2.1. Study Design and PRISMA Adaptation

This narrative review was conducted following PRISMA principles adapted for non-systematic reviews to enhance transparency in reporting. No review protocol was registered.

### 2.2. Study Aims and Type

This study, guided by a narrative review, aimed to review, synthesize, and describe various methods, tools, and outcome measures for monitoring antimicrobial use in outpatients in HICs, incorporating published literature and input from experts working in this field.

### 2.3. Definitions

In this review, outpatient settings refer to primary healthcare clinics, outpatient departments of hospitals, and community pharmacies. In line with the WHO terminology, antimicrobial use refers to both medicine-level and clinical-level antimicrobial use (see definitions in Supplement file) and includes both antibiotics and other antimicrobials such as antifungals, antivirals, and antiprotozoals. When applying the AWaRe classification, we used the term ‘antibiotics’ and ‘antibiotic use’ rather than the broader ‘antimicrobials’ and ‘antimicrobial use,’ in alignment with WHO terminology. High-income countries were identified as per the World Bank Group classification for the financial year 2024 (1 July 2023–30 June 2024) [17].

### 2.4. Search Strategy

We searched the MEDLINE database using Ovid and Embase for articles describing data sources, methodologies, and survey protocols for monitoring antimicrobial use in outpatients in HIC published from inception to 14 August 2023. The screening focused on keywords related to high-income countries, antimicrobial agents, outpatient care settings, dispensing points, utilization patterns, data collection methods, and standardized metrics. The detailed search methodology is available in Supplementary S1.

Two researchers (AK and MC) manually screened the reference lists from the search of all included articles for relevant studies. Web searches using Google were performed to identify cross-references, and confirm links to registries mentioned in the articles. We contacted researchers with expertise in monitoring the use of medicines, identified through their collaboration with WHO and involvement in related technical projects, to obtain information about studies from their countries. We conducted a specific search for studies using AWaRe as one of the outcome measures to identify additional studies.

### 2.5. Inclusion Criteria

We included all quantitative studies written in English that reported numeric data on antimicrobial use in outpatient settings in HICs based on any method or any outcome measures. We excluded studies related to inpatient settings, studies aggregating the total use of antimicrobials for both hospital and outpatients without ability to disaggregate, qualitative studies on behavior or attitudes of stakeholders regarding AMU.

### 2.6. Data Collection and Extraction

We reviewed the articles included to characterize them based on methodology and study characteristics using predefined criteria. Specifically, for each study, we identified the methodology (routine surveillance database or survey) and the reported outcomes were noted and tabulated. Data were extracted using a predefined template and cross-checked for accuracy. Extracted variables included study characteristics (country, setting, year), data source type, patient demographics, disease focus, and reported outcome measures. The details for study characterization and reported outcome measures are provided below.

### 2.7. Method Characterization

We characterized the methods based on whether the data were retrieved using routine surveillance with healthcare databases or involved primary data collection through surveys.

### 2.8. Study Characterization

We characterized the studies on antimicrobial use based on methods used (i.e., routine surveillance, surveys), type of database for routine surveillance, study year (i.e., before 2000; 2000–2009; 2010- onwards), and WHO Region.

Identification of variables, data sources, and outcome measures

To characterize how the information was reported from each study, we examined (a) sample or population characteristics (including demographic details based on age, sex, ethnicity, socioeconomic status, prescriber characteristics, and geospatial information), (b) disease or treatment indications, (c) time periods (e.g., annual data, trends, patterns, and seasonal variations), and (d) outcome measures, including those based on AWaRe.

### 2.9. Risk of Bias Assessment

No formal risk of bias assessment was conducted, as the aim was descriptive mapping of methods rather than evaluation of intervention effects.

### 2.10. Data Analysis

We conducted a descriptive analysis using predefined categories based on our data extraction framework. Studies were classified by data collection method (routine surveillance or survey), surveillance database type, study period, and WHO Region. Key characteristics, such as patient group, disease focus, healthcare level, country, data source, and outcomes, were tabulated for all studies. A narrative synthesis summarized the strengths and limitations of each data source, highlighting methodological diversity and identifying gaps in data collection practices.

## 3. Results

### 3.1. Search Results

**Figure 1:**
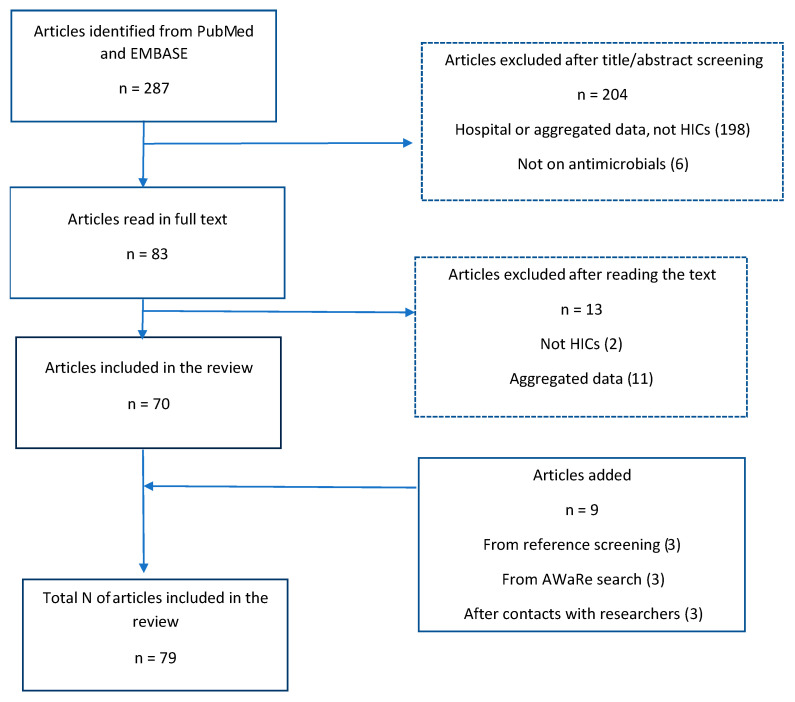
Flow diagram summary of the paper selection process.

The literature search of PubMed and EMBASE identified 287 studies. After title and abstract screening, we selected 83 potentially relevant studies for full-text screening. Of these, we excluded 13 either because they reported aggregated data, or the study was not from HICs. We added nine more studies after screening references (*n* = 3), reaching out to 29 experts (*n* = 3), and conducting a targeted search for AWaRe outcome measures (*n* = 3). The selection process resulted in a total of 79 studies fulfilling the criteria for synthesis in this review (Figure 1).

### 3.2. Study Characteristics

Of the 79 studies, 3 were conducted before 2000, while 76 were conducted from 2000 onwards. These studies originated from four WHO regions that include HICs (Table 1).

### 3.3. Methods and Data Sources

**Figure 2:**
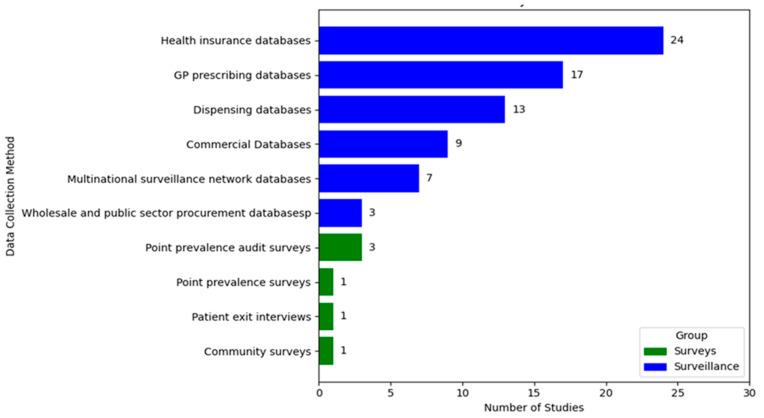
Number of studies on antimicrobial use in outpatients in HICs by data collection method.

Overall, 73 studies used surveillance databases and 6 studies used structured surveys (Figure 2). Tables S1–S7 in Supplementary S2 summarize all the studies according to their key characteristics, patient target group and disease, methods, and outcome measures.

We identified six types of databases, listed in order from most to least frequently used: health insurance databases, prescribing databases, dispensing databases, commercial databases, multinational surveillance network databases, and wholesale and public sector procurement databases.

Among the surveys, we identified four types, listed in order from most to least frequently used: point prevalence audit surveys (i.e., focus on antimicrobial use related to a particular diagnosis, e.g., urinary tract infections or respiratory infections), point prevalence general surveys (i.e., capture antimicrobial use across all indications within a population or setting), patient exit interviews accompanied by prescription review, and community survey.

### 3.4. Studies Utilizing Routine Healthcare Databases

Overall, 73 out of the 79 studies (92%) utilized routinely collected data to measure antimicrobial use among outpatients. They leveraged existing datasets, gathered through healthcare systems, electronic health records, and surveillance programs. Tables S1–S6 in Supplementary S2 present all the studies according to their surveillance databases for data collection. The tables summarize studies’ key characteristics, such as country, patient target group and disease, healthcare system level, database, and outcome measures.

Studies utilizing dispensing databases

A total of 13 studies used dispensing databases, with 1 study from Sweden conducted before 2000 [18], 1 study from Spain from 2000 to 2009 [19], and 11 studies from 2010 onwards [20,21,22,23,24,25,26,27,28,29,30]. These 11 studies were from Canada (*n* = 2) [20,21], Denmark (*n* = 2) [22,23], Norway (*n* = 2) [24,25], Belgium (*n* = 1) [26], the Netherlands (*n* = 1) [27], Portugal (*n* = 1) [28], Spain (*n* = 1) [29], and the USA (*n* = 1) [30]. Table S1 describes all identified studies that used dispensing databases, summarizing their target population, country, and reported measures. All dispensing databases contained the details of medicines dispensed, and 31% (4/13) also contained information on patients’ indications [24]. The common measures reported from dispensing databases include Defined Daily Doses/1000 inhabitants/day (DID) (*n* = 9), antimicrobial prescriptions/1000 inhabitants/day (PID) (*n* = 5), prescribing by age group (e.g.) (*n* = 4), infection-specific prescribing (*n* = 4), and regional variations in prescribing (*n* = 3). One more recent study also reported the total and relative prescribing by AWaRe, Access-to-Watch index, and Amoxicillin index [29].

#### 3.4.1. Studies Utilizing Health Insurance Databases

Overall, 24 studies using health insurance databases were identified: 1 study from Australia published before 2000 [31], 2 studies from 2000 to 2009, 1 from UK [32] and 1 from Italy [33], and the rest, 21, after 2010, including studies in Japan (*n* = 4) [34,35,36,37], Republic of Korea (*n* = 3) [38,39,40], Australia (*n* = 2) [41,42], Belgium (*n* = 2) [43,44], Italy (*n* = 2) [45,46], France (*n* = 2) [47,48], Canada (*n* = 1) [49], Croatia (*n* = 1) [50], Denmark and Germany (*n* = 1) [51], Finland (*n* = 1) [52], New Zealand (*n* = 1) [53], USA (*n* = 1) [54]. Table S2 illustrates all identified studies with health insurance databases, indicating their target population and disease, country, and reported measures. Insurance databases provided patient-level characteristics, including indication, age, sex, ethnicity, socioeconomic class [53], or prescriber characteristics such as specialty [37]. Studies that used insurance databases commonly reported metrics such as DID (*n* = 15), PID (*n* = 7), and Days of Therapy (DOT) (*n* = 6), and stratified the prescribing patterns by age (*n* = 11), class of antimicrobial prescribed (*n* = 7), and AWaRe (*n* = 2).

#### 3.4.2. Studies Utilizing GP Prescribing Databases

A total of 17 studies used GP prescribing databases for studying antimicrobial use, all from after the year 2010, were conducted in the Netherlands (*n* = 3) [55,56,57], UK (*n* = 3) [58,59,60], Canada (*n* = 2) [61,62], France (*n* = 2) [63,64], Australia (*n* = 1) [65], Ireland (*n* = 1) [66], Italy (*n* = 1) [67], Portugal (*n* = 1) [68], Saudi Arabia (*n* = 1) [69], Sweden (*n* = 1) [70], and Switzerland (*n* = 1) [71]. Table S3 summarizes all identified studies utilizing GP database along with details of target population, country, and the reported outcome measures. GP databases had the advantage of providing indications or relevant investigations related to the prescription of antimicrobials. The most commonly used metrics were antibiotic prescriptions either per 1000 consultations, patient-years, or registered patients (*n* = 11) compared with rarely used DID (*n* = 2). GP prescribing databases were used to gain insight into the antimicrobial prescribing practices by indication like respiratory tract infections (RTI) [65] and urinary tract infection (UTI) [25]. One study covered the adherence to clinical guidelines in terms of choice of antibiotics and another one studied duration of treatment. Five studies looked at antibiotic prescribing by AWaRe [62,64,67,69,71].

#### 3.4.3. Studies Using Commercial Databases

We identified nine studies, all from the year 2010 onwards that were from Canada (*n* = 3) [72,73,74], USA (*n* = 2) [75,76], France (*n* = 1) [77], Germany (*n* = 1) [78], Romania (*n* = 1) [79], and Switzerland (*n* = 1) [80]. These studies used commercial healthcare information databases that offer a collection of healthcare information, encompassing sales, de-identified prescription data, medical claims, and electronic medical records at a cost. One study presented the use of antibiotics by AWaRe [80]. The identified studies utilizing commercial databases with dispensing, GP, and sales records are presented in Table S4.

#### 3.4.4. Wholesale and Public Sector Distribution Databases

Databases of public sector healthcare facilities/central pharmaceutical supply services have been utilized to study antimicrobial use in few countries, including Malta [81] and Trinidad and Tobago [82]. Wholesale databases have been used for outpatient settings in Estonia and Malta [81,83]. The study conducted in Malta and published in 2011 evaluated AMU for the years 2007–2009 based on two datasets—licensed wholesaler distributor records and government pharmaceutical services [81]. All three studies reported antimicrobial use by DID. Their key characteristics are presented in Table S5.

#### 3.4.5. Multinational Surveillance Network Database

An example for multinational surveillance for AMU in outpatient settings is the European Surveillance of Antimicrobial Consumption Network (ESAC-Net), coordinated by the European Centre for Disease Prevention and Control (ECDC). ESAC-Net collects and analyzes data from 27 European Union (EU) countries and two European Economic Area (EEA) countries, disaggregated by outpatient and inpatient sectors [84]. The data reported to ESAC-Net has been used in various studies comparing antimicrobial use (AMU) across Europe [85,86,87,88,89]. Out of these, one study was published between 2000 and 2009 [85]. A retrospective study using ESAC-Net data evaluated outpatient AMU for 2017 and longitudinally (1997–2017) [90]. Another study used a Bayesian model to assess trends and changes in AMU [91]. Commonly reported ESAC-Net measures for annual antimicrobial use (Table S6) are total antimicrobial use and pattern in each country, trends in antimicrobial use expressed in DIDs, over the years, total use and pattern of each class of antibiotic in different EU/EEA countries and DIDs, including trends of antimicrobial use in outpatients over several years.

### 3.5. Patient Surveys

We identified six studies from HICs where AMU data in outpatients were collected through two types of surveys targeting either health professionals (i.e., point prevalence general surveys and point prevalence audit surveys for specific clinical condition) or community members (i.e., patient exit interviews accompanied by prescription review, and community survey). The six identified patient surveys are presented in Table S7.

#### 3.5.1. Point Prevalence Surveys and Point Prevalence Audits Surveys

There were four European studies utilizing PPS and PPAS methodology to assess antimicrobial use in outpatients in HIC from 2010 onwards. One PPS study was conducted in Spain using quality indicators to assess the general antibiotic prescribing for outpatients [92]. Three prescription audits were performed as a series of multinational PPAS between 2020 and 2022 in several European countries looking at GP-specific prescribing for RTIs [93,94,95].

#### 3.5.2. Patient Interview and Prescription Review

One study based on patient interviews in combination with a prescription review in Greece (before 2000) looked at the most prescribed antibiotics in outpatients and patients’ understanding about the use of prescribed medicines including antibiotics [96].

#### 3.5.3. Community Surveys

Another survey methodology used in Europe involved community surveys with the public, such as the Eurobarometer-Antibiotics survey regularly conducted by the European Commission to assess and monitor EU citizens’ awareness, attitudes, and behaviors regarding antibiotic use and AMR [97]. These surveys measure antimicrobial use among consumers, estimating the percentage of respondents who received antibiotics in the past year. Surveys were conducted in 2009, 2013, 2016, 2018, and 2022 [97].

### 3.6. Outcome Measures

We identified several outcome measures on antimicrobial use (Box 1). Routinely collected medicine-level data reported the outcomes often by Defined Daily Doses per 1000 inhabitants per day (DID), Prescriptions per 1000 inhabitants per day (PID), and Days of Therapy (DOT) as total or relative use stratified by AWaRe, antimicrobial classes, etc. Surveys collected outcome measures such as the proportion of patients prescribed antibiotics, the proportion of patients with specific syndromes or indications treated with specific antibiotics (including by AWaRe classification), age-specific prescribing patterns, and whether the antibiotics were dispensed with or without a prescription. We further provide definitions and suggested applications for these common metrics in Table 2 to support interpretation and comparability across settings.

Box 1 **Summary list of outcome measures**

Surveillance of medicine-level data by using databases

     - Total use

     - Relative use

- AWaRe classification: stratified by Access, Watch, and Reserve categories
- Antimicrobial classes: specific classes of antimicrobials
- Spectrum of action: narrow-spectrum vs. broad-spectrum antibiotics
- Age-specific prescribing
- Regional (subnational) prescribing

Surveys with patient-level data

- Percentage of all patients prescribed antibiotics
- Age-specific prescribing
- Diagnosis-specific prescribing patterns: percentage of patients with specific syndromes or indications treated with antibiotics, including by AWaRe classification
- Antibiotic use with or without prescription

We also analyzed the strengths and limitations of each data collection method in terms of the type, accuracy, and applicability of antimicrobial use information they provide (Table 3).

#### Outcome Measure by AWaRe Classification

AWaRe as an outcome measure was identified in 9 of our 79 studies, conducted in Switzerland (*n* = 2) [71,80], Canada (*n* = 1) [62], Denmark and Germany (*n* = 1) [51], Italy (*n* = 1) [67], France (*n* = 1) [64], Japan (*n* = 1) [36], Saudi Arabia (*n* = 1) [69], and Spain (*n* = 1) [29]. The AWaRe measure was reported utilizing routine surveillance databases (i.e., dispensing, insurance, GPs, commercial). Table 4 provides an overview of these studies, including country, year of publication, data source type, and AWaRe indicators reported (e.g., Access-to-Watch index, Amoxicillin index, AWaRe proportions). For example, the ECDC multinational surveillance network reported the outpatient antimicrobial use data by AWaRe for all 29 participating European countries (Table S7). The patterns of antimicrobial use by AWaRe were presented in charts, allowing for a comparative analysis and facilitating interventions tailored to each country’s needs [98].

### 3.7. Registries

We compiled a list of registries maintained by many government or autonomous bodies in HICs (Table 5). These registries have datasets, dashboards, and web-based applications presenting the data on antimicrobial use in outpatient settings in HICs either publicly or internally. Table 5 provides an overview of national drug-use registries in HICs, summarizing their data granularity (ATC level, age and sex, and sector), access type (open dashboards or proprietary), and official online links.

## 4. Discussion

This narrative review mapped methods used to monitor antimicrobial use in outpatients to better understand the primary care sector where most of the use occurs. We found out that data on antimicrobial use was collected to describe existing practices [82], compare AMU practices at individual facility, subnational [99], national [91], or prescriber specialty levels [37], track AMU changes over time [88,89,90], inform intervention strategies [90], and evaluate impact of interventions [42,100]. Identified studies used either surveillance data or structured surveys, each offering varying detail and unique strengths and limitations. Public health decision-makers can select methods based on data availability and monitoring goals.

Our review showed that HICs predominantly utilize the method of routine surveillance of antimicrobial use in outpatients, extracting data from existing healthcare databases, as in 73 out of 79 studies. These databases varied, encompassing options including pharmacy dispensing data, GP prescribing records, wholesale distribution records, insurance claims data, and commercial databases. The choice of databases differed across countries, depending on the surveillance objectives, health system structure, regulatory frameworks, and data reporting mechanisms. In Europe, HICs participated in the multinational network (ESAC-Net), submitting disaggregated data on antimicrobial use for the outpatient and hospital sectors to a central agency (ECDC). This data is collected annually and made publicly available through a comprehensive dashboard, facilitating benchmarking, transparency, and accessibility. Similarly, our website search identified that 18 HICs maintained public online registries of medicine use in outpatients, providing dashboards and web-based applications with detailed overviews. While all identified registries are maintained by public agencies, permission is often required to access individual-level data for external analysis. All these tools, originating from routine monitoring, not only enhanced public access to data but also supported healthcare professionals, policymakers, and citizens in monitoring and optimizing antimicrobial use on a regular basis.

Surveys were less employed to monitor antimicrobial use in primary care (in 6 out of 79 studies). Point prevalence surveys (PPS) and point prevalence audits surveys (PPAS) provided snapshots of antimicrobial use at specific points in time, offering insights into patient-level use patterns and compliance with guidelines. The individual surveys we identified varied in their methodologies, diseases, and target populations in terms of demographics. Of six surveys, three reported on general antimicrobial use, while three focused on specific disease-related antimicrobial use (i.e., respiratory infections). PPS offered a cross-section of antimicrobial use practice estimated total antimicrobial use or relative antimicrobial patterns. PPAS were used to estimate antimicrobial use, and the survey instrument could be customized as per the outcome of interest or for a specific indication. Mixed approaches involving patient interview and prescription review can complement the surveys, offering insight into factors influencing antimicrobial use and knowledge. Our search identified one community survey in HICs, Eurobarometer—Antibiotics, which was a regular source of data on EU citizens’ behavior regarding antibiotic use, including over-the-counter use of antibiotics, providing insights for public health monitoring and policy making. Yet, surveys are time- and resource-intensive, and the risk of selection bias together limit their wider use in practice.

In this narrative review, we looked specifically at measures reporting the antimicrobial use by AWaRe. The WHO AWaRe classification provides a standardized framework to monitor and optimize antibiotic use, including in primary care where most antibiotics are prescribed. The only existing global target is the 2024 UNGA commitment that at least 70% of national antibiotic use should come from the Access group [101]. We found that routinely collected data reported outcomes using DID, PID, or DOT as total use and stratified by AWaRe groups, and antimicrobial classes. For example, dispensing data in Spain assessed Access antibiotic use in children [29], while insurance databases in Japan tracked trends in Access and Watch group use [36]. Similar analyses were conducted using national or commercial databases in Italy, France, Switzerland, Canada, and Saudi Arabia [62,64,67,69,71]. Switzerland also used commercial databases to estimate antimicrobial use according to AWaRe [80]. The ESAC-Net reports showed a population-weighted mean of 60% Access antibiotic use in the EU/EEA in 2022, increasing slightly to 61% in 2023 (range: 42–75%), with ten countries already meeting or exceeding the EU target of 65% Access use by 2030 [102,103]. The 2025 GLASS Report showed that in 2022, 35 of the 60 reporting countries (58%) met the 60% Access group target, and 19 out of the 60 reporting countries (32%) met the 70% Access group target at national level. [4] Beyond database studies, a prescription survey in Swiss long-term care facilities reported a 22% reduction in total antibiotic use and a 45% drop in Watch group use over five years, following guideline implementation and multidisciplinary quality circles [104].

Despite WHO’s endorsement of AWaRe as a global stewardship and surveillance tool, our review found limited uptake, with only nine studies reporting AWaRe-based outcomes. This low number should also be interpreted in the context that AWaRe was only introduced in 2017, leaving a relatively short timeframe for integration into national surveillance systems and research protocols. Several other barriers may explain this gap. First, incomplete mapping between ATC codes and AWaRe categories complicates automated classification, particularly for combination products. Second, Reserve-class antibiotics are often underreported or aggregated with other categories, limiting visibility of critical agents. Third, integration of AWaRe metrics into national surveillance systems remains inconsistent, as many countries rely on legacy indicators such as DID or DOT without stratification by AWaRe. Additional challenges include lack of awareness among prescribers and insufficient technical capacity to adapt existing databases. Addressing these barriers requires harmonized coding standards, updates to electronic health records, and targeted training. AWaRe-based indicators identified by Funiciello et al. demonstrate feasibility in outpatient settings, suggesting that progressive adoption of metrics for quality assessment is achievable through targeted implementation and international collaboration [105].

Most studies identified focused on a single data source to report AMU data. However, triangulating data from different sources, including routine databases and surveys, enables a better understanding of the processes and behavior of AMU in outpatients. Such a mixed-methods approach, combining diverse data sources, provides deeper insights into the complex dynamics of AMU in outpatients. The UK TARGET (Treat Antibiotics Responsibly, Guidance, Education and Tools) initiative exemplified the importance of comprehensive data collection. By leveraging four distinct databases on prescriptions, resistance patterns, and demographics of patients and prescribers to implement audit and feedback [106,107], TARGET triangulated quantitative data to develop effective antimicrobial stewardship interventions that reduce inappropriate AMU and AMR. Antimicrobial Use and Resistance in Australia (AURA) system is another example of combining datasets, which serves as the national framework for monitoring AMU and AMR in Australia. AURA draws data from various sources, including insurance data from the Pharmaceutical Benefits Scheme, GP prescribing data from Medicine Insight, the Australian Group on Antimicrobial Resistance (AGAR), and the Australian Passive AMR Surveillance (APAS). Since its inception, AURA has published five reports, with the most recent one released in 2023 [108,109,110], providing critical insights into AMU and AMR trends, resistance mechanisms, and prescribing practices.

Although this manuscript focuses on HICs, it is important to consider how these findings might, or might not, apply to LMICs. Many LMICs face constraints, including limited centralized databases and fewer resources such as staff, time, and funding for data collection and analysis. These challenges can make implementation difficult or lead to different outcomes compared to HICs. Nevertheless, there are practical strategies to address these barriers. LMICs can start by integrating existing health information systems and electronic medical records into a unified platform, prioritizing essential indicators. Regional collaborations and shared infrastructures can help reduce costs, while open-source database solutions and cloud-based platforms offer scalable options that minimize upfront investment. Capacity-building initiatives, such as training data managers and IT staff, and phased implementation approaches can further support sustainability. Highlighting these opportunities demonstrates that, despite current limitations, LMICs can progressively strengthen data systems and benefit from these methods. Our study may have suffered from limitations. First, all articles and websites reviewed were in English so some content may have been missed with the search. Excluding non-English publications may have reduced the comprehensiveness and context of the findings, given the linguistic diversity across HICs. Second, we did not conduct a formal systematic review but opted for a narrative review [111]. We chose this approach for its ability to include a wide range of sources beyond the published literature (i.e., websites of healthcare organizations and research networks, gray literature), provide a richer contextual understanding, and be more practical compared to a systematic review. While articles could have been missed, we covered a broader prospective. Previous studies on antimicrobial use with a broader policy focus rather than clinical practice successfully applied narrative reviews, including the identification of AWaRe indicators for appropriate antibiotic use and the exploration of antimicrobial stewardship in hospital settings [105,112].

Third, excluding LMICs comparators limits global generalizability; to address this, we plan a future synthesis combining our reviews of data sources from both HICs and LMICs. Additional limitations include the invisibility of private-sector dispensing, unrecorded over-the-counter antibiotic sales [2], and potential misclassification between medicines prescribed, dispensed, and consumed. These gaps could be mitigated by triangulating registry data with point prevalence surveys and population surveys such as Eurobarometer. Finally, the COVID-19 pandemic may have influenced registry structures and reporting practices, potentially compromising timeliness and completeness of data. Going forward, we recommend a focused set of strategies to strengthen antimicrobial use monitoring and stewardship. First, establishing general and country-specific indicators and targets for antimicrobial use will guide policy and stewardship efforts, ensuring interventions are tailored to local contexts. Second, enhanced data integration, including systems that integrate data from various sources, such as electronic health records, prescription databases, and surveillance systems, could provide a comprehensive view of antimicrobial use patterns. Third, implementing real-time monitoring tools to track antimicrobial use and resistance patterns would enable timely interventions and adjustments to policies and stewardship programs. Fourth, advanced analytics and machine learning algorithms could identify trends, predict outbreaks, and assess the impact of interventions on antimicrobial use and resistance. Fifth, collaboration between healthcare providers, researchers, and policymakers could help develop and implement effective antimicrobial use monitoring strategies. Finally, engaging in international collaborations, such as WHO GLASS-AMU, to share data, best practices, and research findings would enhance the global effort to combat AMR by consolidating analyses and interpretations.

## Figures and Tables

**Table 1:** Overview of study characteristics.

Category	Subcategory	No. Studies (%)
by study year	1991–2000	3 (3.8)
	2000–2009	4 (5.1)
	2010–2023	72 (91.1)
by WHO Region	WHO European Region (34 of 53 Member States are HICs)	53 (67.1)
	WHO Region of the Americas (13 of 35 Member States are HICs)	13 (16.5)
	WHO Western Pacific Region (10 of 38 Member States are HICs)	12 (15.2)
	WHO Eastern Mediterranean Region (6 of 22 Member States are HICs)	1 (1.3)
	WHO African Region (0 of 47 Member States are HICs)	0 (0)
	WHO South-East Asia Region (0 of 11 Member States are HICs)	0 (0)
by data collection method	Data retrieved from routine medicine monitoring databases	73 (92.4)
	Data collected through surveys	6 (7.6)

**Table 2:** Common metrics for monitoring antimicrobial use: definitions and suggested applications.

Metric	Definition	Denominator	Suggested Use-Cases
DID	Number of DDDs dispensed or prescribed per 1000 inhabitants per day	Population size	Best for cross-country comparisons; limited for pediatrics due to adult-based WHO DDD standards
DOT	Number of days a patient receives an antimicrobial, regardless of dose	Patient-days or encounters	Recommended by CDC for stewardship; reflects treatment duration independent of dose
PID/PrID	Number of prescriptions issued per 1000 inhabitants per day	Population size	Useful for assessing prescriber behavior and appropriateness audits
SID	Number of standard units (e.g., tablets, capsules) dispensed	Population size or pharmacy data	Helpful when DDD calculation is not feasible; often used in procurement or wholesale data
AWaRe Stratified Use	Proportion of total antimicrobial consumption classified by WHO AWaRe categories (Access, Watch, Reserve)	Total antimicrobial use (DDD, DOT, or prescriptions)	Essential for monitoring alignment with WHO stewardship targets; supports policy benchmarking and prioritization

**Table 3:** Strengths and limitations of different data sources [24,29,37,38,45,54,55,66].

Methods	Description	Limitations
Routine Surveillance		
Dispensing databases	Offers a closer picture of patient antimicrobial use compared to prescribing, procurement, or wholesale data.	Difficult to obtain from the private sector; does not provide information on actual patient use or prescriber behavior.
Health insurance databases	Provides patient-level data on antimicrobial use disaggregated by patient demographics, geographic characteristics, and indications for use.	Coverage of only insured populations and reimbursed antimicrobials; potential gaps in administrative data.
GP prescribing databases	Contains patient characteristics, diagnosis, prescriptions, dose duration, indications, and co-prescribed medicines.	Limited by the number of participating GPs; may not represent the entire region or nation; prescribed antibiotics may not be dispensed.
Wholesale and public sector distribution databases	Provides aggregated data on the distribution and procurement of antibiotics from wholesalers and public health sectors.	May not capture patient-level details or actual usage; limits insights into prescribing behaviors and patient adherence.
Surveillance networks databases	Uses national sales or reimbursement data to monitor antibiotic consumption across countries.	Heterogeneity in healthcare systems among different countries may hinder direct comparisons.
Surveys		
Point Prevalence Surveys (PPS)	Customizable data collection tools tailored to specific study parameters.	Resource-intensive; may fail to capture dynamic trends unless conducted repeatedly.
Point Prevalence Audit Surveys (PPAS)	Examines consultation and management characteristics of patients with specific conditions.	Resource-intensive; may fail to capture dynamic trends unless conducted repeatedly.
Patient exit interviews	Capture real-time information on prescribing, dispensing (with or without a prescription), and patient understanding after consultations.	Resource-intensive; may suffer from recall and social desirability bias and may not reflect actual antibiotic use or adherence.
Community Surveys	Provides consumer-level data closely reflecting actual antimicrobial use in outpatients.	Resource-intensive, may suffer from representativeness and bias issues.

**Table 4:** Summary of studies reporting AWaRe metrics.

Country and Year of Publication	Reference	Methodology (Type of Database/Survey)	AWaRe Indicators
Spain, 2021	[29]	National pharmacy dispensing database	AWaRe proportions (Access, Watch, Reserve)
Japan, 2020	[36]	National health insurance claims database	AWaRe proportions
Denmark and Germany, 2021	[51]	GP prescribing database (electronic medical records)	AWaRe proportions
Canada, 2021	[62]	National claims database	AWaRe proportions
France, 2021	[64]	National claims database (IQVIA)	AWaRe proportions
Italy, 2022	[67]	Pedianet pediatric EMR database	Antibiotic index, Access-to-Watch index, Amoxicillin-to-Co-amoxiclav index
Saudi Arabia	[69]	Hospital outpatient pharmacy dispensing database	AWaRe proportions
Switzerland	[71]	Survey of outpatient prescribing practices	AWaRe proportions
Switzerland	[80]	Wholesale distribution database	AWaRe proportions

**Table 5:** National registries/databases for drug use in different high-income countries and reported outcomes.

No.	Country and Reference	Registries/Database	Data Granularity	Data Access
1	Australia, [31,42]	Pharmaceutical Benefits Scheme (PBS) [https://www.pbs.gov.au/pbs/home] https://www.pbs.gov.au/pbs/home (accessed on 7 November 2025 Medicine Insight [https://www.nps.org.au/medicine-insight/using-medicineinsight-data] (accessed on 7 November 2025)	ATC Level: 5 Age/sex stratification available Sector stratification available	PBS—Open dashboard Medicine Insight—Proprietary
2	Belgium [26,43,44]	Farmanet—community pharmacy reimbursed dispensations [https://www.riziv.fgov.be/nl/statistieken/geneesmiddel/Paginas/Statistieken-geneesmiddelen-apotheken-farmanet.aspx] (accessed on 7 November 2025)	ATC Level: 3 and 5 Age/sex stratification available Sector stratification available	Restricted access—data request needed, not fully open dashboard
3	Croatia [50]	Agency for Medicinal Products and Medical Devices (HALMED) [https://www.halmed.hr/en/O-HALMED-u/Osnovni-podaci-i-dokumenti/HALMED-i-korisnici/] (accessed on 7 November 2025)	ATC Level: 5 Age/sex stratification not available Sector stratification not available	Reports only, no interactive dashboard; proprietary for detailed data [halmed.hr]
4	Denmark [22,23]	National Prescription Registry [https://sundhedsdatastyrelsen.dk/borger/om-sundhedsdata/sundhedsdatastyrelsens-registre] (accessed on 7 November 2025)	ATC Level: 4 and 5 Age/sex stratification available Sector stratification available	Aggregated data via eSundhed (open); individual-level requires application
5	Finland [52]	Finnish Prescription Registry/Kelasto [https://raportit.kela.fi/ibi_apps/WFServlet?IBIF_ex=NIT137AL&YKIELI=E] (accessed on 7 November 2025)	ATC Level: 5 Age/sex stratification available Sector stratification available	Open dashboard (Kelasto statistical reports)
6	France [47,48]	National Health Insurance (SNDS) [https://www.snds.gouv.fr/SNDS/Accueil] (accessed on 7 November 2025)	ATC Level: 3 and 4 Age/sex stratification available Sector stratification available	Mixed: Open data (Open Medic) + proprietary for detailed SNDS access
7	Japan [37]	National Database of Health Insurance Claims and Specific Health Checkups (NDB) [https://ndb6nc.ncgm.go.jp/eng/outline/index.html] (accessed on 7 November 2025)	ATC Level: 3 Age/sex stratification available Sector stratification available	Open data portal available (NDB Open Data)
8	Norway [25]	Norwegian Prescription Database (NorPD) [https://www.norpd.no/] (accessed on 7 November 2025)	ATC Level: 5 Age/sex stratification available Sector stratification available	Open aggregated reports; detailed data requires application
9	Netherlands [27]	Foundation for Pharmaceutical Statistics (SFK) [https://www.hiv-monitoring.nl/en/research-using-our-data/datakoppelingen/sfk] (accessed on 7 November 2025)	ATC Level: 5 Age/sex stratification available Sector stratification available	Proprietary (data sold via SFK or partners like SpotOnInsights)
10	New Zealand [53]	Pharmaceutical Collection https://www.tewhatuora.govt.nz/for-health-professionals/data-and-statistics/nz-health-statistics/national-collections-and-surveys/collections/pharmaceutical-collection (access on 7 November 2025)	ATC Level: 5 Age/sex stratification available Sector stratification available	Open summary stats via Ministry of Health; detailed data restricted
11	Portugal [68]	INFARMED [https://www.infarmed.pt/web/infarmed-en/about-infarmed] (accessed on 7 November 2025)	ATC Level: 3 Age/sex stratification limited Sector stratification not available	Reports only, no open dashboard; proprietary for detailed data
12	Republic of Korea [38,39,40]	Health Insurance Review and Assessment Service (HIRA) [https://www.hira.or.kr/eng/main.do]	ATC Level: 4 Age/sex stratification available Sector stratification available	Open dashboard for claims data (HIRA public portal)
13	Sweden [15,18]	Swedish Prescribed Drug Register [https://www.socialstyrelsen.se/en/statistics-and-data/registers/national-prescribed-drug-register/] (accessed on 7 November 2025) Swedish eHealth Agency [https://www.ehalsomyndigheten.se/languages/english/welcome-to-the-swedish-ehealth-agency/] (accessed on 7 November 2025)	ATC Level: 3 Age/sex stratification available Sector stratification available	Reports available, detailed data requires application
14	United Kingdom [59,60]	Clinical Practice Research Datalink (CPRD)—MHRA [https://cprd.com/] (accessed on 7 November 2025)	ATC Level: 3 Age/sex stratification available Sector stratification available	Proprietary (requires license and approval)

## Data Availability

No new data were created or analyzed in this study. Data sharing is not applicable to this article.

## Supplementary Materials

The following supporting information can be downloaded at: https://www.mdpi.com/article/10.3390/antibiotics14111161/s1, Supplementary S1: Search history; Supplementary S2: Summary of studies monitoring outpatient antimicrobial use in HICs; Table S1: Studies utilizing dispensing databases; Table S2: Studies utilizing national insurance databases; Table S3: Studies utilizing GP prescribing databases; Table S4: Studies using commercial databases; Table S5: Studies using wholesale and public sector distribution databases; Table S6: Protocols based on data from ESAC-Net; Table S7: Patient surveys on antimicrobial use in primary care.

## Abbreviations

The following abbreviations are used in this manuscript:

Abbreviation	Description
AMR	Antimicrobial Resistance
AMU	Antimicrobial Use
AWaRe	Access, Watch, Reserve (WHO Classification Of Antibiotics)
WHO	World Health Organization
GLASS	Global Antimicrobial Resistance and Use Surveillance System
GLASS-AMU	GLASS Module For Antimicrobial Use
HICs	High-Income Countries
DID	Defined Daily Doses Per 1000 Inhabitants Per Day
DOT	Days of Therapy
PID	Prescriptions Per 1000 Inhabitants Per Day
RTI	Respiratory Tract Infections
UTI	Urinary Tract Infections
PBS	Pharmaceutical Benefits Scheme (Australia)
NDB	National Database of Health Insurance Claims and Specific Health Checkups (Japan)
NorPD	Norwegian Prescription Database
SFK	Foundation for Pharmaceutical Statistics (Netherlands)
INFARMED	Portuguese National Authority of Medicines and Health Products
CPRD	Clinical Practice Research Datalink (UK)
EMRALD	Electronic Medical Record Administrative Data Linked Database (Canada)
ODB	Ontario Drug Benefit (Canada)
IPCRN	Irish Primary Care Research Network
ESAC-Net	European Surveillance of Antimicrobial Consumption Network
ECDC	European Centre for Disease Prevention and Control
AGAR	Australian Group on Antimicrobial Resistance
APAS	Australian Passive AMR Surveillance
AURA	Antimicrobial Use and Resistance in Australia
THIN	The Health Improvement Network (UK)
EMRPC	Electronic Medical Records Primary Care (Ontario, Canada)
SID	Standard Units Per 1000 Inhabitants Per Day
PrID	Prescriptions Per 1000 Inhabitants Per Day
PPS	Point Prevalence Survey
PPAS	Point Prevalence Audit Survey
