# Supplementary material for: Overview of Methods for Assessing Antimicrobial Use in Outpatient Settings in High-Income Countries: A Narrative Review
Source: Antibiotics (Basel). 2025 Nov 16;14(11):1161. doi: 10.3390/antibiotics14111161 (PMC12649155; doi:10.3390/antibiotics14111161)
Supplement: Supplementary file 1 [file antibiotics-14-01161-s001.zip › antibiotics-3919589-supplementary.pdf]

antibiotics MDPI

Supplementary materials

Supplementary S1: Search history

A preliminary search was conducted through the MEDLINE and Embase databases from inception to August 14, 2023.

Ovid Search

Search All Ovid Journals

1 (Andorra? or "Antigua and Barbuda" or Antigua or Barbuda or Aruba or Australia* or Austria* or Bahama or Bahamas or Bahrain or Barbados or Belgium or Belgian or Bermuda or British Virgin Islands or Brunei Darussalem or Canada or Canadian or Cayman Islands or Channel Islands or Chile or Chilean or Croatia or Croatian or Curcao* or Cyprus or Czech or Czech Republic or Denmark or Estonia or Estonian or Faroe Islands or Finland or Finnish or France or French or French Polynesia or French Polynesian or Germany or German or Gibraltar or Greece or Greek or Greenland or Guam or Hong Kong or Hong Kong SR or Hungary or Hungarian or Iceland or Icelandic or Ireland or Irish or "Isle of Man" or Israel or Israeli or Italy or Italian or Japan or Japanese or "Republic of Korea" or "Korean Republic" or Kuwait or Kuwaiti or Latvi* or Liechtenstein or Lithuania or Luxembourg or Macao* or Malta or Monaco or Nauru or Netherlands or Dutch or "New Caledonia" or "Caledonian" or "New Caledonian" or "New Zealand" or "Northern Mariana Islands" or Norway or Norwegian or Oman or Panama or Poland or Polish or Portugal or Portuguese or "Puerto Rica" or "Puerto Rican" or Qatar or Qatari or Romania* or "San Marino" or Saudi Arabia or Arab or Seychelles or Singapore or Singaporian or "Sint Maarten" or "Slovak Republic" or Slovak or Sloveni* or Spain or Spanish or "St. Kitts and Nevis" or "St. Kitts & Nevis" or "St. Martin" or Sweden or Swedish or Swiss or Switzerland or Taiwa* or Trinidad or Tobago or "Trinidad and Tobago" or "Turks and Caicos" or "Turks and Caicos Islands" or "United Arab Emirates" or UAE or "United Kingdom" or Britain or British or "United States" or USA or American or Uruguay or "Virgin Islands").mp. [mp=title, abstract, full text, caption text] 5986410

2 ("high-income countries" or "high income countries" or "developed countries").mp. [mp=title, abstract, full text, caption text] 129741

3 1 or 2 6002802

4 (Antimicrobial or Antibiotic or antibacterial or anti-microbial or anti-bacterial).mp. [mp=title, abstract, full text, caption text] 483416

5 (primary health or primary care or community care or primary healthcare or outpatient or OPD or family practice or general practice or general practitioner or physician).mp. [mp=title, abstract, full text, caption text] 1348116

6 (pharmacy or dispensing or retail pharmacy or drug retailer).mp. [mp=title, abstract, full text, caption text] 213266

7 5 or 6 1477863

8 (drug utilization or drug utilisation or practice pattern or overuse or underuse or consumption or misuse or inappropriate or overpresc* or underpresc* or over-use).mp. [mp=title, abstract, full text, caption text] 858130

9 3 and 4 and 7 and 8 22455

10 (questionnaire or survey or protocol or monitoring or surveillance).mp. [mp=title, abstract, full text, caption text] 3121715

11 9 and 10 15079

12 (defined daily dose or DDD 1000 day).mp. [mp=title, abstract, full text, caption text] 3289

13 9 and 12 419
14 ((questionnaire or survey or protocol) not hospital).mp. [mp=title, abstract, full text, caption text] 1509327
15 ((questionnaire or survey or protocol) not inpatient).mp. [mp=title, abstract, full text, caption text] 2404537
16 9 and 15 9028
17 12 and 16 126

Embase search

Embase

Session Results

....

No.	Query Results	Results	Date
#14.	#11 AND #12 NOT 'inpatient' NOT icu	263	14 Aug 2023
#13.	#11 AND #12	322	14 Aug 2023
#12.	'defined daily dose' OR ddd	13,829	14 Aug 2023
#11.	#9 AND #10	3,445	14 Aug 2023
#10.	questionnaire OR survey OR protocol OR monitoring	4,643,565	14 Aug 2023
#9.	#3 AND #4 AND #7 AND #8	10,083	14 Aug 2023
#8.	'drug utilization' OR 'drug utilisation' OR 'practice pattern' OR overuse OR underuse OR consumption OR misuse OR inappropriate OR overpresc* OR underpresc* OR 'over use'	867,946	14 Aug 2023
#7.	#5 OR #6	2,695,390	14 Aug 2023
#6.	pharmacy OR dispensing OR 'retail pharmacy' OR 'drug retailer'	1,151,541	14 Aug 2023
#5.	'primary health' OR 'primary care' OR 'community care' OR 'primary healthcare' OR outpatient OR opd OR 'family practice' OR 'general practice' OR 'general practitioner' OR physician	1,616,891	14 Aug 2023
#4.	antimicrobial OR antibiotic OR antibacterial OR 'anti microbial' OR 'anti bacterial'	1,161,369	14 Aug 2023
#3.	#1 OR #2	40,114,430	14 Aug 2023
#2.	'high-income countries' OR 'high income countries' OR 'developed countries'	56,578	14 Aug 2023
#1.	andorra? OR 'antigua and barbuda'/exp OR 'antigua	40,110,617	14 Aug 2023

and barbuda' OR 'antigua'/exp OR antigua OR barbuda OR 'aruba'/exp OR aruba OR australia* OR austria* OR 'bahama'/exp OR bahama OR 'bahamas'/exp OR bahamas OR 'bahrain'/exp OR bahrain OR 'barbados'/exp OR barbados OR 'belgium'/exp OR belgium OR 'belgian'/exp OR belgian OR 'bermuda'/exp OR bermuda OR 'british virgin islands'/exp OR 'british virgin islands' OR (('british'/exp OR british) AND ('virgin'/exp OR virgin) AND ('islands'/exp OR islands)) OR 'brunei darussalem' OR (('brunei'/exp OR brunei) AND (darussalem) OR 'canada'/exp OR canada OR 'canadian'/exp OR canadian OR 'cayman islands'/exp OR 'cayman islands' OR (('cayman'/exp OR cayman) AND ('islands'/exp OR islands)) OR 'channel islands'/exp OR 'channel islands' OR (channel AND ('islands'/exp OR islands)) OR 'chile'/exp OR chile OR 'chilean'/exp OR chilean OR 'croatia'/exp OR croatia OR croatian OR curcao* OR 'cyprus'/exp OR cyprus OR czech OR 'czech republic'/exp OR 'czech republic' OR (czech AND republic) OR 'denmark'/exp OR

denmark OR 'estonia'/exp OR estonia OR estonian OR 'faroe islands'/exp OR 'faroe islands' OR (faroe AND ('islands'/exp OR islands)) OR 'finland'/exp OR finland OR 'finnish'/exp OR finnish OR 'france'/exp OR france OR 'french'/exp OR french OR 'french polynesia'/exp OR 'french polynesia' OR (('french'/exp OR french) AND ('polynesia'/exp OR polynesia)) OR 'french polynesian'/exp OR 'french polynesian' OR (('french'/exp OR french) AND ('polynesian'/exp OR polynesian)) OR 'germany'/exp OR germany OR 'german'/exp OR german OR 'gibraltar'/exp OR gibraltar OR 'greece'/exp OR greece OR 'greek'/exp OR greek OR 'greenland'/exp OR greenland OR 'guam'/exp OR guam OR 'hong kong'/exp OR 'hong kong' OR (hong AND kong) OR 'hong kong sr' OR (hong AND kong AND ('sr'/exp OR sr)) OR 'hungary'/exp OR hungary OR 'hungarian'/exp OR hungarian OR 'iceland'/exp OR iceland OR icelandic OR 'ireland'/exp OR ireland OR 'irish'/exp OR irish OR 'isle of man'/exp OR 'isle of man' OR 'israel'/exp OR israel OR 'israeli'/exp OR israeli OR 'italy'/exp OR italy OR 'italian'/exp OR italian OR 'japan'/exp OR japan OR 'japanese'/exp OR japanese OR 'republic of korea'/exp OR 'republic of korea' OR 'korean republic' OR 'kuwait'/exp OR kuwait OR 'kuwaiti'/exp OR kuwaiti OR latvi* OR 'liechtenstein'/exp OR liechtenstein OR 'lithuania'/exp OR lithuania OR 'luxembourg'/exp OR luxembourg OR macao* OR 'malta'/exp OR malta OR 'monaco'/exp OR monaco OR 'nauru'/exp OR nauru OR 'netherlands'/exp OR netherlands OR 'dutch'/exp OR dutch OR 'new caledonia'/exp OR 'new caledonia' OR 'caledonian' OR 'new caledonian'/exp OR 'new caledonian' OR 'new zealand'/exp OR 'new zealand' OR 'northern mariana islands'/exp OR 'northern mariana islands' OR 'norway'/exp OR norway OR 'norwegian'/exp OR norwegian OR 'oman'/exp OR oman OR 'panama'/exp OR panama OR 'poland'/exp OR poland OR 'polish'/exp OR polish OR 'portugal'/exp OR portugal OR 'portuguese'/exp OR portuguese OR 'puerto rica' OR 'puerto rican'/exp OR 'puerto rican' OR 'qatar'/exp OR qatar OR 'qatari'/exp OR qatari OR romania* OR 'san marino'/exp OR 'san marino' OR 'saudi arabia'/exp OR 'saudi arabia' OR (('saudi'/exp OR saudi) AND ('arabia'/exp OR arabia)) OR 'arab'/exp OR arab OR 'seychelles'/exp OR seychelles OR 'singapore'/exp OR singapore OR singaporian OR 'sint maarten'/exp OR 'sint maarten' OR 'slovak republic'/exp OR 'slovak republic' OR slovak OR sloveni* OR 'spain'/exp OR spain OR 'spanish'/exp OR spanish OR 'st. kitts and nevis'/exp OR 'st. kitts and nevis' OR 'st. kitts & nevis' OR 'st. martin'/exp OR 'st. martin' OR 'sweden'/exp OR sweden OR 'swedish'/exp OR swedish OR 'swiss'/exp OR swiss OR 'switzerland'/exp OR switzerland OR taiwa* OR trinidad OR tobago OR 'trinidad and tobago'/exp OR 'trinidad and tobago' OR 'turks and caicos' OR 'turks and caicos islands'/exp OR 'turks and caicos islands' OR 'united arab emirates'/exp OR 'united arab emirates' OR uae OR 'united kingdom'/exp OR 'united kingdom' OR 'britain'/exp OR britain OR 'british'/exp OR british OR 'united states'/exp OR 'united states' OR 'usa'/exp OR usa OR 'american'/exp OR american OR 'uruguay'/exp OR uruguay OR 'virgin islands'

The appendix is an optional section that can contain details and data supplemental to the main text—for example, explanations of experimental details that would disrupt the flow of the main text but nonetheless remain crucial to understanding and reproducing the research shown; figures of replicates for experiments of which representative data is shown in the main text can be added here if brief, or as Supplementary data. Mathematical proofs of results not central to the paper can be added as an appendix.

Supplementary S2: Summary of studies monitoring outpatient antimicrobial use in HICs

Table S1: Studies utilizing dispensing databases

Study	Target Group	Disease	Level	Country	Data Source	Reported Measures

Mölstad & Cars, 1999 [18]	General Population	Generic	National	Sweden	Sweden's county-level pharmacy dispensing records by Apoteket AB's pharmacy sales statistics and regional prescription monitoring programs	DDD/1000 inhabitants/day (DID), antibiotic use for RTI, seasonal variation, antibiotic use by age group (0–4, 5–12, 13–29, 30–64, >65 years), antibiotic use by counties
Campos et al., 2007 [19]	General Population	Generic	National	Spain	Reimbursement data for ambulatory care by Spanish Agency for Medicines and Healthcare Products and sales data by International Medical Statistics Health	DID, Prescriptions per 1000 Inhabitants per Day (PID)
Schwartz et al., 2019 [20]	Older Adults (65+)	Generic	National	Canada	Electronic Medical Record Administrative Linked Database (EMRALD) and Ontario Drug Benefit (ODB) dispensing database	PID, antibiotic prescribing rates and dispensing trends, proportion of antibiotic prescriptions per consultation
Glass-Kaastra et al., 2014 [21]	General Population	Generic	National	Canada	Outpatient antimicrobial use data by Canadian provincial databases 2000–2010	DID, PID, prescribing rates, antibiotic prescribing trends across provinces, yearly trends
Aabenhus et al., 2016 [22]	General Population	Respiratory infections	National	Denmark	Danish national prescribing registries 2004–2013	DID, PID, prescribing rates, duration of antibiotic treatment, trends over time
Veimer Jensen et al., 2021 [23]	Elderly Population	Generic	National	Denmark	Danish National Prescription Registry - reimbursed prescriptions collected at outpatient pharmacies	DID, PID, antibiotic utilization in elderly patients
Blix & Høye, 2021 [24]	General Population	Generic	National	Norway	Norwegian Drug Wholesale Statistics, Norwegian Prescription Database (NorPD) 2010-2017	DID, total systemic antibiotic sales, prescriptions issued, antibiotic use for RTI, age-related trends
Haugom et al., 2021 [25]	General Population	Urinary Tract Infections	National	Norway	Norwegian primary care consultation and prescribing records (2006–2015) Registry-based study	DIDs, UTI-related antibiotic prescribing trends, frequency of consultations for UTIs, choice of antibiotics used for UTI
Dillen et al., 2025 [26]	Paediatric Population	Generic	National	Belgium	Belgian Farmanet - pharmaceutical dispensing database in community pharmacies	DID, antibiotic use trends in children, dispensing frequency by age, changes in antibiotic selection over time
de Jong et al., 2019 [27]	General Population	Generic	National	Netherlands	Foundation for Pharmaceutical Statistics (SFK) 2006–2014 Retrospective, annual outpatient antibiotic use data analysis	Antibiotic treatment episodes (prescriptions), duration of antibiotic use, antibiotic switch rates
Gomes et al., 2015 [28]	General Population	Generic	National	Portugal	INFARMED's national pharmaceutical records (2004–2014)	DID, regional variations in antibiotic use, yearly trends in outpatient antibiotic utilization

Calle-Miguel et al., 2022 [**29**]	Paediatric Population	Generic	Regional	Spain (Asturias)	Billing data of the containers dispensed in the pharmacy offices run by the National Health System	DID, overall dispensing and by AWaRe, Access-to-Watch index, Amoxicillin index
King et al., 2021 [**30**]	General Population	Generic	National	USA	IQVIA Total Patient Tracker for dispensed antibiotic prescriptions from retail pharmacies	Number of patients dispensed antibiotic prescriptions, overall antibiotic dispensing rates and by agent, class, patient age, state, and prescriber specialty, monthly comparison

Table S2: Studies utilizing national insurance databases

**Study**	**Target Group**	**Disease**	**Level**	**Country**	**Data Source & Study Methodology**	**Reported Measures**
Birkett et al., 1991 [**31**]	General Population	Generic	National	Australia	Australian Pharmaceutical Benefits Scheme pensioner data (1987–1989) and market research data from a private company	DDD per 1000 pensioners per day (DDD/1000 per day), prescribing trends for upper respiratory tract infections, otitis media, sinusitis
Davey et al., 2008 [**32**]	General Population	Generic	National	UK	European Surveillance of Antimicrobial Consumption (ESAC) project data (1997–2005)	DID, PID, prescribing trends across UK administrations
Gagliotti et al., 2009 [**33**]	General Population	Urinary Tract Infections	Regional	Italy (Emilia-Romagna)	Reimbursement data from the Emilia-Romagna Region (2007) and sales data from IMS Health	DID, prescribing trends for urinary tract infections
Kinoshita et al., 2019 [**34**]	Paediatric Population	Generic	National	Japan	National health claims database (2013–2016)	Days of therapy (DOT) per infectious disease-related visit, prescribing trends for paediatric patients
Okubo et al., 2020 [**35**]	Paediatric Population	Generic	National	Japan	National health claims database (2015–2016)	Days of Therapy (DOT), total and broad-spectrum antibiotic use
Okubo et al., 2021 [**36**]	Paediatric Population	Generic	National	Japan	National health claims database (2013–2018)	Days of Therapy (DOT), trends in antibiotic prescription rates across clinics over time, AWaRe classification and Amoxicillin Index.
Ono et al., 2020 [**37**]	Dental Patients	Generic	National	Japan	National health claims database and Specific Health Checkups (NDB) (2015–2017)	DID, Proportion of prescriptions by antibiotic class, total, by class, and dentists vs GPs
Song et al., 2018 [**38**]	Paediatric Population	Generic	National	South Korea	Health Insurance Review and Assessment Service (HIRA) (2010-2014)	DID, Proportion of outpatient prescriptions for antibiotic, trends in antibiotic class selection over time, comparison of prescribing patterns by age group

Study	Target Group	Disease	Level	Country	Data Source & Study Methodology	Reported Measures
Park et al., 2017 [**39**]	General Population	Generic	National	South Korea	Health Insurance Review and Assessment Service (HIRA) (2007–2014)	DIDs, prescribing trends by age group and antibiotic classes
Yoon et al., 2015 [**40**]	General Population	Generic	National	South Korea	Health Insurance Review and Assessment Service (HIRA) (2008–2012)	DIDs, prescribing trends for different antibiotic classes
Gadzhanova & Roughead, 2020 [**41**]	Paediatric Population	Generic	National	Australia	Australian Pharmaceutical Benefits Scheme (PBS) data (2013)	Dispensing rates per 1,000 children, Proportion of children receiving antibiotics, prescribing by age, antibiotic class, and single vs. multiple antibiotics
Contreras et al., 2023 [**42**]	General Population	Generic	National	Australia	Australian Pharmaceutical Benefits Scheme (PBS) (2019–2020)	Prescriptions and dispensations per 1,000 inhabitants, total antibiotic prescriptions before and after policy implementation, proportion of appropriate supply of original prescriptions and repeat prescriptions, prescribing patterns for specific antibiotics
Coenen et al., 2014 [**43**]	General Population	Generic	National	Belgium	Belgian Farmanet – reimbursement and dispensing database (2002–2009)	DID, Number of packages per 1,000 inhabitants per day, Number of treatments per 1,000 inhabitants per day, trends in outpatient antibiotic use over time, comparison of different measurement approaches (DDD vs. packages vs. treatments).
Struyf et al., 2020 [**44**]	Dental Patients	Generic	National	Belgium	Reimbursement data from the Belgian National Institute for Health and Disability Insurance (INAMI/RIZIV) (2010–2016)	DID, Packages per 1,000 inhabitants per day (PID, DDD and packages per prescriber, Trends in antimicrobial prescribing rates over time, total and by specific antibiotics
Russo et al., 2018 [**45**]	General Population	Generic	National	Italy (Campania)	Campania health databases - Reimbursement pharmacy records (2016)	DIDs, prescribing trends by socioeconomic and sociodemographic factors
Cangini et al., 2021 [**46**]	General Population	Generic	National	Italy	Italian National Health System databases: Farmanet and Flusso Informativo delle Prestazioni Ambulatoriali (FIPA) for dispensed and reimbursed medicines (2013–2018)	DID, proportion of broad-spectrum vs. narrow-spectrum antibiotics, by AWaRe, regional and seasonal variations
BARA et al., 2022 [**47**]	General Population	Generic	National	France	French National Health Data System (SNDS)	Prescriptions per 1,000 consultations, antibiotic prescribing rate per 1,000 patients

Study	Target Group	Disease	Level	Country	Data Source & Study Methodology	Reported Measures
Bernier et al., 2014 [48]	Elderly Population	Generic	National	France	French National Health Insurance databases (2000–2010)	DID, prescriptions per 1,000 inhabitants, trends and age-specific prescribing patterns
Saatchi et al., 2021 [49]	General Population	Generic	National	Canada	British Columbia Centre for Disease Control data (2000–2018)	Prescriptions per 1,000 inhabitants, antibiotic dispensing rates, long-term trends and paediatric prescribing patterns
Vojvodić & Daus Šebeďak, 2018 [50]	General Population	Urinary Tract Infections	National	Croatia	Croatian national health insurance database (2005–2014)	DID, Prescriptions per 1,000 inhabitants trends in antibiotic use for urinary infections
Scholle et al., 2024 [51]	Infant Population	Generic	National	Denmark & Germany	Danish healthcare registries & German claims database (2004–2016)	Prescriptions per 1,000 inhabitants, AWaRe, Median time to first antibiotic prescriptions, cross-country comparison of early-life antibiotic prescribing
Pyörälä et al., 2022 [52]	Adult Population	Generic	National	Finland	Finnish national reimbursement databases (Kelasto) (2008–2019)	DID, Cost per prescription, trends in outpatient antibiotic use, cost analysis, age-specific prescribing patterns
Williamson et al., 2016 [53]	General Population	Generic	National	New Zealand	New Zealand national pharmaceutical database (2006–2014)	DID, trends in antibiotic consumption, disparities in prescribing by age, sex, and ethnicity, seasonal variation in prescribing
Olesen et al., 2018 [54]	Older adults (≥65 years)	Generic	National	USA	Medicare administrative claims data (2011–2015)	Prescription claims per 1,000 beneficiaries per year, appropriate vs. inappropriate prescribing rates, antibiotic-specific trends

Table S3: Studies utilizing GP prescribing databases

Study	Target Group	Disease	Level	Country	Data Source & Study Methodology	Reported Measures
van den Broek d'Obrenan et al., 2014 [55]	General Population	Generic	National	Netherlands	Dutch primary care electronic health records. (2007–2010)	Antibiotic prescriptions per 1,000 consultations., prescribing rates per infectious disease episode, antibiotic choice per diagnosis
van der Velden et al., 2020 [56]	General Population	Various infections	Regional	Netherlands (Utrecht)	Dutch primary care antibiotic surveillance system - patients' medical files for 2017 extracted from 44 GP practices by two Dutch	Total number of prescribed antibiotics per 1000 registered patients, Prescribing percentages for episodes of RTI

Study	Target Group	Disease	Level	Country	Data Source & Study Methodology	Reported Measures
					companies: Medworq and INSZO-STIZON	
Haeseker et al., 2012 [**57**]	General Population	Generic	National	Netherlands	Dutch Registration Network Family Practices (RNH) (2000–2009)	Antibiotic prescriptions per 1,000 patient-years, trends in antibiotic prescribing by age group, by antibiotic classes, association between antibiotic use and adverse drug events
Smith et al., 2018 [**58**]	General Population	Generic	National	UK	Large computerized general practitioner database 'The Health Improvement Network (THIN)' (1995–2011)	Antibiotic prescriptions per 1,000 patient-years, trends in prescribing across different age groups, prescribing rate by syndrome, % of consultations with antibiotic prescription, % of antibiotic prescriptions for the condition that adhered to the recommended antibiotics recommended treatment length
Gulliford et al., 2020 [**59**]	General Population	Serious bacterial infections	National	UK	UK Clinical Practice Research Datalink (CPRD) (2002–2017)	Antibiotic prescriptions per 1,000 patient-years, antibiotic prescribing rates per infection
Sun & Gulliford, 2019 [**60**]	General Population	Generic	National	UK	UK Clinical Practice Research Datalink (2014–2017)	Antibiotic prescriptions per 1,000 patient-years, trends in antibiotic prescribing across different age groups, gender differences, prescribing indications, narrow-spectrum vs b8road spectrum antibiotics
Schwartz et al., 2020 [**61**]	General Population	Generic	Regional	Canada	Electronic Medical Records Primary Care database (Ontario, Canada) (2011–2016)	Antibiotic prescribing rates per 100 patient–physician encounters, rates of unnecessary antibiotic prescriptions overall and age group, prescribing trends by condition and age group
Edwards et al., 2023 [**62**]	General Population	Generic	Regional	Canada	Newfoundland and Labrador provincial pharmacy network database (2017–2021)	DID, Prescription rate per 1,000 population per year, trends in antimicrobial use, duration of prescriptions, AWaRe, prescribing patterns for specific antibiotics
Soudais et al., 2021 [**63**]	Male Patients	Urinary Tract Infections	National	France	French general practice electronic database (PRIMEGE/MEDISEPT) (2012–2017)	Antibiotic prescriptions per 1,000 consultations, UTI consultation frequency, diagnostic approach, antibiotic selection, treatment duration, bacteriology

Study	Target Group	Disease	Level	Country	Data Source & Study Methodology	Reported Measures
Trinh et al., 2020 [64]	Paediatric Population	Generic	National	France	IQVIA's EPPM database (2015–2017)	Antibiotic prescription rates per 100 visits, trends in antibiotic prescribing for respiratory tract infections, broad-spectrum antibiotic use, AWaRe, prescribing differences between GPs and paediatricians
Bernardo et al., 2019 [65]	General Population	Influenza-like illness	National	Australia	MedicineInsight dataset (2015–2017)	Influenza-like illness (ILI) consultation rates per 1,000 consultations, antibiotic prescribing rates for ILI cases, prescribing trends over time, variability in antibiotic selection across patient groups, and season
Galvin et al., 2015 [66]	General Population	Generic	National	Ireland	Irish Primary Care Research Network (IPCRN) (2012–2013)	DDD per 1000 inhabitants/day, ESAC drug-specific quality indicators for outpatient antibiotic use, total antimicrobial use (DDD) vs. quinolone use (DDD) by month, number of quinolone prescriptions by age group and se1ason
Barbieri et al., 2022 [67]	Paediatric Population	Generic	National	Italy	Pedianet database (2019–2021)	Antibiotic prescription rates per 100 visits, AWaRe classification of prescribed antibiotics, AWaRe classification, antibiotic index, Access-to-Watch index
Ramalhinho et al., 2012 [68]	General Population	Generic	National	Portugal	INFARMED outpatient antibiotic prescription database (2000–2009)	DID, trends in antibiotic consumption, regional variations, shifts in prescribing patterns for specific antibiotic classes
Alshareef et al., 2023 [69]	General Population	Generic	National	Saudi Arabia	Electronic medical records from 24 primary care and dental clinics (2020)	Number of antibiotic prescriptions, DID, AWaRe, adherence to clinical guidelines
Cronberg et al., 2020 [70]	General Population	Generic	Regional	Sweden	Kronoberg Infection Database in Primary Care (KIDPC) (2006–2014)	Antibiotic prescribing rates per 1000 inhabitants per year, relative risk of antibiotic prescribing (out-of-hours vs. in-hours), mean annual change in prescribing rates, gender, age and diagnosis-based variations in prescribing patterns
Martínez-González et al., 2020 [71]	General Population	Generic	National	Switzerland	Electronic medical records from primary care (2008–2020)	Antibiotic prescriptions per 1,000 consultations, antibiotic prescribing rates by clinical indication, prescribing trends over time, most frequently prescribed antibiotics, AWaRe classification proportions, demographic and socioeconomic associations with prescribing rates

Table S4: Studies using commercial databases

Study	Target Group	Disease	Level	Country	Data Source & Study Methodology	Reported Measures
Schwartz et al., 2019 [**72**]	Older Adults (65+)	Generic	National	Canada	IQVIA Xponent antibiotic database and Ontario population-wide outpatient antimicrobial dispensing database (2016–2017)	Mean number of antibiotic prescriptions per physician, antibiotic prescribing rate for physicians, duration of antibiotic therapy
Kitano et al., 2021 [**73**]	General Population	Generic	Regional	Canada (Ontario)	Electronic Medical Records Primary Care database in Ontario (EMRPC) (2011–2016)	Days of Therapy (DOT), Total antibiotic prescriptions per patient visit, association between total antibiotic volume and unnecessary prescribing inter-physician variability
Knight et al., 2022 [**74**]	General Population	Generic	National	Canada	IQVIA CompuScript database (2014–2020)	Days of Therapy (DOT), impact of COVID-19 on antibiotic dispensing trends, changes in antibiotic selection
Hicks et al., 2015 [**75**]	General Population	Generic	National	USA	QuintilesIMS Xponent database (US outpatient prescriptions) (2011)	Antibiotic prescriptions per 1,000 persons, prescribing rates by provider specialty, geography, patient age and sex
Bizune et al., 2024 [**76**]	General Population	Generic	National	USA	IQVIA National Prescription Audit dataset (US retail pharmacy data) (2019–2022)	Number of prescriptions, monthly antibiotic prescriptions before and during COVID-19, trends in azithromycin use,<br>seasonal variations
Trinh et al., 2018 [**77**]	General Population	Generic	National	France	IQVIA SDM database (2009–2016)	Prescriptions per 1,000 inhabitants per day (PrID), DID, Packages per 1,000 inhabitants per day (PID), Standard units per 1,000 inhabitants per day (SID).<br>Discrepancies between DID and PrID trends, antibiotic prescribing patterns
Kern & Kostev, 2021 [**78**]	General Population	Acute respiratory infections	National	Germany	Disease Analyzer database (2015–2019)	Prevalence of antibiotic prescriptions, association between antibiotic prescriptions and specific diagnoses (bronchitis, sinusitis, pharyngitis), prescribing differences between general and paediatric practices
Popescu et al., 2013 [**79**]	General Population	Generic	National	Romania	IMS Health Romania sales data (2012)	DID, ESAC-Net quality indicators for outpatient use of antibiotics - inappropriate broad-spectrum antibiotic use
Plüss-Suard et	General Population	Generic	National	Switzerland	IQVIA sales data (2018–2023)	DID, AWaRe classification trends, interrupted time series analysis, post-Covid antibiotic consumption trends, regional variations in

Study	Target Group	Disease	Level	Country	Data Source & Study Methodology	Reported Measures
al., 2024 [80]						prescribing, shifts in Access vs. Watch group antibiotic use

Table S5: Studies using wholesale and public sector distribution databases

Study	Target Group	Disease	Level	Country	Data Source & Study Methodology	Reported Measures
Zarb & Borg, 2011 [81]	General Population	Generic	National	Malta	Wholesale distribution records	DID, trends in ambulatory antibiotic use - total and broad-spectrum vs narrow-spectrum, age-related trends, diagnosis-specific prescribing patterns
Khan et al., 2021 [82]	General Population	Generic	Regional	Trinidad & Tobago	Stock requisition and logbooks from a primary care facility (2011–2018)	Defined Daily Doses (DDD) per 1,000 residents per day, trends over time, most frequently prescribed antibiotics, diagnosis-specific prescribing patterns
Lass et al., 2020 [83]	General Population	Generic	National	Estonia	Estonian national health database (2008 –2018)	DID, prescribing rates, age-related trends, diagnosis-specific prescribing patterns, seasonal variations

Table A6: Protocols based on data from ESAC-Net

Study	Target Group	Disease	Level	Country	Data Source & Study Methodology	Reported Measures
Ferech et al., 2006 [85]	General Population	Generic	Multi-country	Europe	ESAC-Net database (1997–2003)	DID, total antimicrobial use and patterns in each country, diagnosis-specific prescribing patterns
McDonnell et al., 2017 [86]	General Population	Generic	Multi-country	Europe	ESAC-Net database and European Antimicrobial Resistance Surveillance Network (EARS-Net) database	DID, national antimicrobial use rate, national antimicrobial resistance rate diagnosis-specific prescribing patterns
Adriaenssens et al., 2011 [87]	General Population	Generic	Multi-country	Europe	ESAC-Net database (1997-2009)	DID, packages per 1,000 inhabitants per day (PID), prescribing rates, yearly outpatient antibiotic use in 33 European countries, total outpatient antibiotic use, seasonal variation, age-related trends, diagnosis-specific prescribing patterns
Bruyndonckx et al., 2021 [88]	General Population	Generic	Multi-country	EU/EEA	ESAC-Net database (EU/EEA countries)	DID, antibiotic use rates expressed in DIDs for 30 EU/EEA countries and PIDs for 20 EU/EEA countries, age-related trends, diagnosis-specific prescribing

Study	Target Group	Disease	Level	Country	Data Source & Study Methodology	Reported Measures
						patterns, trends in penicillin consumption, seasonal variations, country-level prescribing differences
Versporten et al., 2021 [**89**]	General Population	Generic	Multi-country	EU/EEA	ESAC-Net database (1997-2017)	Antibiotic use rates expressed in DIDs for 30 EU/EEA countries and PIDs for 20 EU/EEA countries, age-related trends, diagnosis-specific prescribing patterns, trends for tetracyclines, sulphonamides, and trimethoprim, country-level variation
Bruyndonckx et al., 2021 [**90**]	General Population	Generic	Multi-country	EU/EEA	European Surveillance of Antimicrobial Consumption Network (ESAC-Net).	DID, Packages per 1,000 inhabitants per day (PID), long-term trends in antibiotic consumption across EU/EEA countries, seasonal variations, country-level prescribing differences, identification of change-points in consumption patterns.
Bruyndonckx et al., 2021 [**91**]	General Population	Various infections	Multi-country	EU/EEA	European Surveillance of Antimicrobial Consumption Network (ESAC-Net)	DID, Packages per 1,000 inhabitants per day (PID), trends in antibiotic consumption across EU/EEA countries, seasonal variations, country-level prescribing differences, identification of change-points in consumption patterns

Table S7: Patient surveys on antimicrobial use in primary care

Study	Target Group	Disease	Level	Country	Data Source & Study Methodology	Reported Measures
March-López et al., 2020 [**92**]	General Population	Generic	Regional	Spain (Barcelona)	Primary healthcare electronic medical records from nine PHC centers (2018)<br><br>Point prevalence survey	Number of antibiotic prescriptions, Adherence to prescribing guidelines based on Outpatient Quality Indicators: Avoidance of antibiotics for viral or self-limiting bacterial infections (acute tonsillitis and sinusitis), Use of first-line antibiotics according to clinical guidelines, Appropriate duration of antibiotic therapy, Diagnostic testing before prescribing antibiotics (group A streptococcal testing before prescribing antibiotics for pharyngitis), Avoidance of broad-spectrum antibiotics when narrow-spectrum options are sufficient,

Study	Target Group	Disease	Level	Country	Data Source & Study Methodology	Reported Measures
						Documentation of antibiotic prescriptions, Follow-up assessment after antibiotic prescription
van der Velden et al., 2021 [**93**]	General Population	Respiratory infections	Multi-country	16 European countries (EU and non-EU)	Data entered into an online data capture system (Research Online) directly or from paper forms (April-May 2020)<br><br>Prospective point prevalence audit survey of management of RTIs (COVID-19)	Number of antibiotic prescriptions per 1,000 consultations, RTI antibiotic prescribing, Point-of-care testing rates
van der Velden et al., 2022 [**94**]	General Population	Respiratory infections	Multi-country	18 European countries (EU and non-EU)	Data entered into an online data capture system (Research Online) directly or from paper forms (January-February 2020)<br><br>Prospective point prevalence audit survey of management of RTIs (COVID-19)	Number of antibiotic prescriptions per 1,000 consultations, RTI antibiotic prescribing, Point-of-care testing rates
Vellinga et al., 2023 [**95**]	General Population	Respiratory infections	Multi-country	13 European countries	Data entered into an online data capture system (Research Online) directly or from paper forms (2020, 2021, 2022)<br><br>Prospective point prevalence audit survey of management of RTIs	Percentage of all patients prescribed antibiotics, disease-specific prescribing trends (bronchitis, pneumonia, tonsilitis, cold, sinusitis), prescribing trends by specific antibiotics
Tzimis et al., 1997 [**96**]	Indigent Patients	Generic	National	Greece	Patients interviews and prescription records review	Patients understanding of antibiotic use, Patients medication adherence, Antibiotic consumption in DDD and cost, Most prescribed antimicrobials
Eurobarometer	General	Generic	EU/EEA	Multiple Countries	Public opinion surveys on antibiotics	Antibiotic use trends, public awareness of antimicrobial resistance, self-medication rates

Study	Target Group	Disease	Level	Country	Data Source & Study Methodology	Reported Measures
Surveys [**97**]	Population					
